# Understanding Neurobehavioural Dynamics: A Close-Up View on Psychiatry and Quantum Mechanics

**DOI:** 10.21315/mjms2019.26.1.14

**Published:** 2019-02-28

**Authors:** Wani Ab Latif, Shadab Ggha

**Affiliations:** Cytogenetics and Molecular Toxicology Laboratory, Section of Genetics, Department of Zoology, Faculty of Life Science, Aligarh Muslim University, Aligarh, 202002, Uttar Pradesh, India

**Keywords:** brain, behaviour, psychiatry, quantum mechanics, drugs

## Abstract

Psychiatric disorders are prevalent throughout the world and causes heavy burden on mankind. Alone in US, billions of dollars are used for treatment purposes annually. Although advances in treatment strategies had witnessed recently, however the efficacy and overall outcome weren’t quite promising. In neurobehavioural sciences, old problems survive through ages and with psychiatric disease, the phenomenon turns intensely complex. While our understanding of brain is mostly based on concepts of particle physics, its functions largely follow the principles of quantum mechanics. The current therapeutics relies on understanding of brain as a material entity that turns to be one of the chief reasons for the unsatisfactory therapeutic outcomes. Collectively, as mankind we are suffering huge loss due to the least effective treatment strategies. Even though we just begin to understand about how brain works, we also do not know much about quantum mechanics and how subatomic particles behave with quantum properties. Though it is apparent that quantum properties like particle and wave function duality coincides with the fundamental aspects of brain and mind duality, thus must share some common basis. Here in this article, an opinion is set that quantum mechanics in association with brain and more specifically psychiatry may take us towards a better understanding about brain, behaviour and how we approach towards treatment.

## Introduction

From the dawn of human existence, consequence of psychiatric diseases is evident throughout. Billions of dollars are used annually for treatment of psychiatry disease. Different treatment methods have been adopted with time; however the outcome is quite unsatisfactory. In brain sciences the problems recognised hundred years ago are still unresolved and with psychiatry it becomes more compounded. With newer advancements, researchers use multiple approaches in understanding brain and its functions. In the quest of discerning brain, little progress was made; though we are still far away from understanding fundamental elements about how brain works. Brain is a complex organ and the principles by which it works through sophisticated mechanisms are even more complex. Individual differences in the brain, behaviour and response to stimuli lead to variations in the onset of mental illness. Wide range of interpretations on how brain works has been published. The need is to recognise the erroneous interpretations which make our efforts on understanding mental illnesses difficult. Advances in the understanding of molecular basis of neuropsychiatric disorders have revealed individual variations in brain, behaviour and the heterogenetic nature of these disorders (1). Efforts to discover and develop new drugs for neuropsychiatric disorders that might revolutionise disease treatment have been relatively unsuccessful (1). Recent advances in the quantum mechanics and biological systems have revolutionised our understanding about the structure and reactivity of small molecular system (2). In present times the need is to embrace every possible treatment strategy to make some positive impact over psychiatric diseases. Recently a mathematical model has been developed based on the established principles of physics. This model includes mechanisms of instincts, emotions, behaviour, concepts, intuition, cognition and imagination leading to the classification of the fundamental notions of opposition between conscious and unconscious (3, 4).

The classical understanding about human consciousness underwent a major shift, as modern understanding bought greater changes. The aim of this article was to acquire an understanding of brain and its complex functions in association with what is known in quantum mechanics. Here, we set an opinion that understanding psychiatry and in large the brain can be helped by bringing it to parallel with what we know in quantum mechanics.

## Classical Understanding

**Figure 1:**
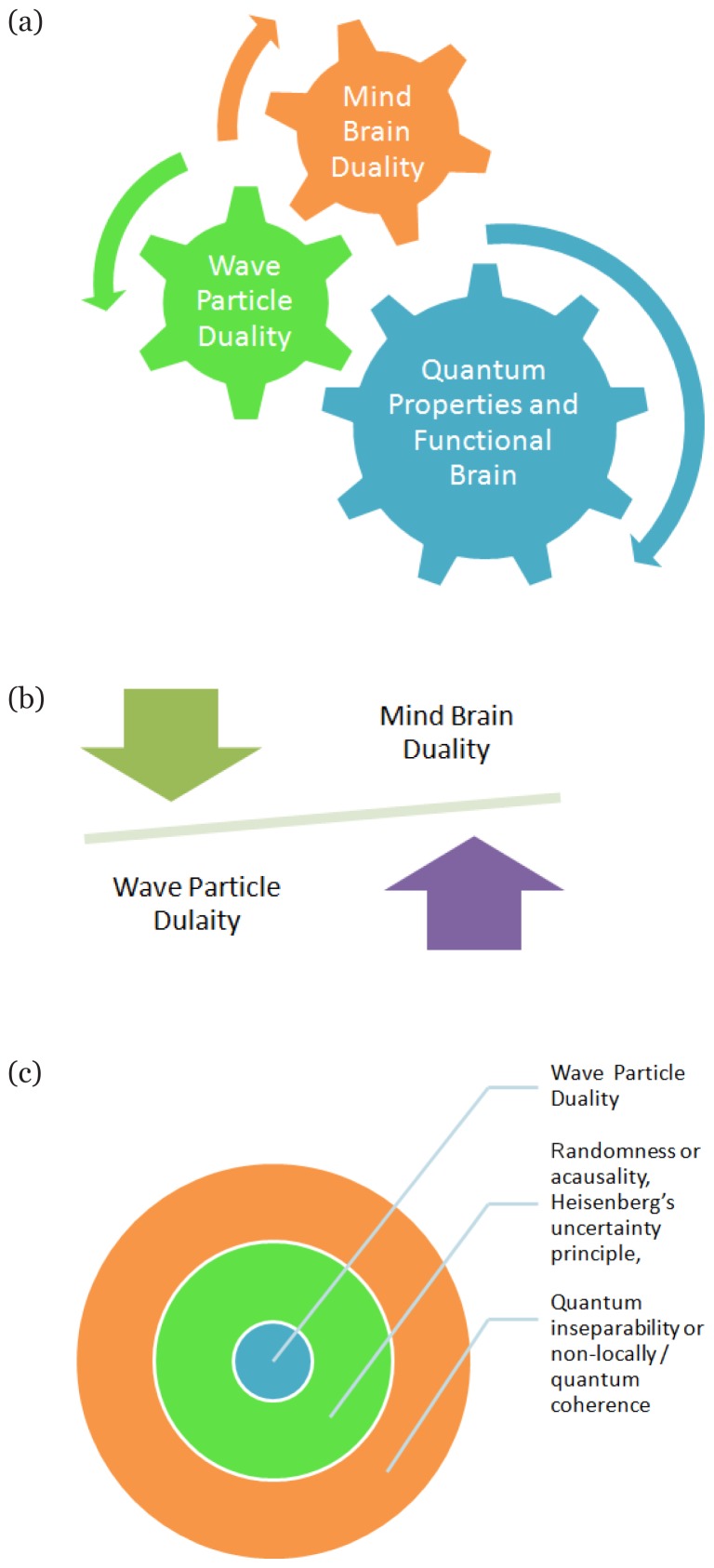
(a) Indicates the quantum properties like wave particle duality and mind brain duality are intertwined for the functional brain, (b) Indicates that for normal functional brain there should be a balance between diverse functions of the brain, while (c) Indicates different properties of quantum mechanics which are contained and consolidated for the functional brain in the real world

Until recently, the understanding of life and more specifically brain and how it functions was based on principles of classical or Newtonian physics (5). With advances in scientific understanding, such principles were known as fundamentally false, largely due to their conflict with the nature of subatomic particles (6). Though classical physics significantly helps in the scientific advancement of mankind during 21st century, and with the passage of time it was found in conflict with the modern understanding about how universe works. It is assumed that a parallel perspective exists between how brain and universe functions. Classical physics promotes the understanding that universe and everything in it is based on material things and interactions between them (7). The modern understanding has almost changed those concepts after the foundation of quantum physics (8). New understanding emphasise on the role of non-physical traits like emitted energy which forms the electromagnetic spectrum (9). This offers an alternative conceptual framework and the scientific description with respect to structural modelling of the brain based on self directed neuroplasticity (5). Our universe is comprised of matter and energy the fundamental properties of which are described by quantum theory (10). Haemeroff and Penrose suggest that conscious experiences play an important role for operation of the laws of universe. Further, they also ascribe that quantum superposition occur within the microtubules of neurons (11). The quantum properties have been repeatedly verified which include wave/particle duality, randomness or acausality, Heisenberg’s uncertainty principle, quantum inseparability or non-locally/quantum coherence (12). Even though these quantum properties are bizarre, strange and counter intuitive, they help in understanding the mysterious nature of consciousness (12). Though there may be different possibilities on how these bizarre functions of the brain could become physical reality and appear in life. The interrelated and overlapping ideas associated with brain in relation to quantum properties are graphically depicted in Figure 1 (a)–(c).

The etiology in the malfunctioning of brain is diverse with such an extent that every psychiatric disorder could be associated with unique predispositions. Psychiatric disorders are complex and the currently used less effective procedures often turn them more debilitated. One of the reasons is heterogeneity in brain diseases and individual differences that make the dysfunctions person specific (13). Disruptions in signals between neurons are often considered responsible for various brain disorders. The signals in brain are carried to their targets through the complex neurocircuit connections by means of specific pathways. Long thread like part of neurons used to conduct impulses from cell body to other cells is called axons. Axons were appeared to be guided by four combined operations like short and long term attraction and short and long term repulsion. These are also called as contact-attraction and chemo-attraction and contact-repulsion and chemo-repulsion (14). Brain consists of billions of neurons which are interconnected by trillions of connections to form hard wired brain circuitry (15). Such brain circuits could be highly variable in their networking by carrying signals based on individual interaction to outside environment. Even the number of these circuits varies with time due to neuroplasticity (16). Individual differences and variable environmental interaction possibly lead to huge variation in neurochemistry. Thus, due to huge diversity of neurocircuit networking, there are chances that the same psychiatric disorder could have multiple origins among humans. By current therapeutics, usually the same treatment methods are used among individuals with different etiology, which is marked with low success rate. One reason could perhaps be due to large number of circuits in brain and inefficiency to find out the exact location of the defective circuit.

## Environment: An Interactive Entity

Neurobehavioural abnormalities and other major diseases develop due to considerable degree of environmental interactions. Such interactions must be considered in the development of therapeutics. Considering heterogeneity among individuals and variable gene environment interaction, the pathways involved in the development of neurobehavioural disorders must be diverse. Understanding such diversity in pathways may help us getting closer towards effective treatments. The present approaches used for treatment of neurobehavioural disorders are mostly ineffective because of little or no consideration to environment. As though environment has significant role in the development of etiology, following the same treatment procedure for individuals living in diverse environments may be one important reason for ineffective outcome. Though it seems obvious that gene environment interaction will not always be harmful, but also helps the organism adapt environmental variations. However, the mechanism of such interactions and how it differs between beneficial and harmful interaction is yet to be understood. In various psychiatric disorders usually there is coincidence of symptoms. Both nature and nurture influences the growth and development which plays significant role in the onset and progression of disease. The type of interaction between nature and nurture is an essential factor that has important role in the final outcome. Thus, varied underlying mechanisms between gene environment interactions may lead to increased variation in behaviour and epigenetic alterations (17).

## Limitations by Classical Understanding

Classical understanding about the world and importantly on biomedical sciences has undergone some major advancement, shortly after its limitations came into the forefront of biology and medicine. Limitations associated with currently used psychiatric drugs are primarily due to our limited knowledge about how brain functions. Certain psychiatric disorders and their symptoms like bipolar depression with delusions and schizophrenia with hallucinations escape our understanding and thus remain as important limitations of classical physics principles. One of the important symptoms associated with number of psychiatric disease is paranormal phenomenon. Understanding such phenomenon by the principles of classical physics perhaps make our route to effective treatment a possibility far distant. In similar way special features by certain individuals like telepathic communications cannot be explained by Newtonian phenomenon. Though attempts are continuously made to reduce the burden of neurobehavioural disorders; however the outcome is far from expected. This is what concerns Cara too about how mental health establishments don’t learn from mistakes and families always pay price. His focus was on how our present medications don’t serve the effective treatment purposes (18). Kinderman was twice elected as chair of the British psychological society’s division of clinical psychology. In his article in scientific American on 17 November 2014, he showed a deep concern while he said “we need whole sale and radical change, not only in how we understand mental health problems, but also in how we design and commission mental health services” (19). A recent research shows that human genomes are extremely personalised, when it was observed that most of the genes exist in different forms within the population (20). In the aforementioned study around four million gene forms were reported in about 400 genomes the researchers analysed (20). This further complicates any advancement in the field of personalised medicine (21).

Burden of psychiatric disorders is enormously increasing with unbearable cost on mankind due to the absence of any effective therapeutics. The question arises what type of universe are we living in which allows the occurrence of paranormal phenomenon and exhibit characteristics similar to that of quantum world. Thus it can be said, that paranormal phenomenon adhered to quantum properties are also expressed in macro-universe; with characteristics of randomness, unpredictability and uncertainty. It is assumed thus, the coincidence about how brain and the universe functions are considerably large thus increases the possibilities for further understanding of less known functions.

Quantum approaches could presumably help us to understand much about hallucinations, delusions and other psychic abnormalities. The complexity of brain can be deduced from the fact that each circuit processes specific type of information (16). The drugs are usually developed to target any imbalance in neurotransmitters in the malfunctioning circuit, which hampers information processing. Neurotransmitter imbalance is considered as one of the important reasons for brain related dysfunctions in which hypothetically many other factors are assumed to have a role. Even though brain has hardwired intercircuitry, an assumption was made earlier that brain could have limited number of circuit ‘highways’ which carry the symptoms (16). A hypothetical question can be asked, could there be a possibility that a drug specifically targeting any symptom can treat that given symptom in all neuropsychiatric disease? Does location of the circuit in different regions of the brain has any role in the complexity of symptoms. It is difficult to exactly locate the defective neurocircuit in the brain consisting over billion neurons. It can be proposed however, that in the developmental course of all neuropsychiatric disorders there must be some etiological predispositions shared by most of them. Working towards understanding these common recourses may lead us towards their effective control.

Paranormal symptoms like hallucination or delusions don’t seem to be the outcome of any alteration in physical tissue. Thus, it seems improbable that the present understanding could help to develop targeted drugs for such paranormal symptoms. Such phenomenon can be understood using an approach which is parallel between paranormal symptoms of mental illness and principles of quantum mechanics. As both these fields share some similarities, the need is to integrate neuropsychiatry and quantum mechanics in order to shift towards better understanding. Newtonian physics emphasises on the material basis of universe, this contention was changed by quantum mechanics which considers the basis of universe as energy. However, it is assumed that giving consideration to only energy aspect in understanding brain cannot be much helpful. Thus, emphasising on the existence of both matter and energy simultaneously can help towards better understanding. Concerning about the importance of both matter and energy Pauli in 1955 said:

“The only acceptable point of view appears to be the one that recognises both sides of reality—the quantitative and the qualitative, the physical and the psychical—as compatible with each other and can embrace them simultaneously” (22).

Our current understanding about life and biological processes is the foundations for designing therapeutics. This understanding contains some important limitations that also integrate in the current therapeutics. The need is to understand the coexistence of matter and energy which is important and alters most of the bimolecular interactions. Thus, studying interaction between matter and energy may help in better understanding of neuropsychiatric disorders and the ways to tackle them.

Earlier an understanding was developed that brain is a static organ without any power of cellular regeneration. This view was later changed in 21 century with the discovery of specific property of brain called neuroplasticity. It has been observed that brain has the characteristic property of modifying its organisation which alters its function due to individual life experiences and brain thoughts, a property that is called neuroplasticity (23). After this discovery, it is been assumed that brain can be modified towards a healthy growth even during old age by thoughts and specific exercises (24, 25). There is significant preliminary data showing mindful awareness modulate the activity of specific brain regions, e.g., prefrontal cortex (5). Contrary to this, classical physics rely mostly on assumption that everything is determined before birth by the interaction between mindless tiny elements giving no consideration to our mindfulness and focussed intentions. These discoveries showing time and again that brain functions are not limited to the principles of Newtonian physics and thus act as existing forces for joining neuro-behaviour and quantum mechanics.

## Environmental Interaction and Variability in Epigenetic Changes

A new rising field of science, epigenetics shows a marked effect on the development of brain. Epigenetics is the study of heritable changes in gene activity which is not caused by changes in DNA sequence (26). The process of epigenetic changes starts at the time of foetal development in the womb of mother. The experiences a mother passed to foetus during her pregnancy leads to the diverse kinds of epimarks. Experience of stressful periods by mother can decrease the efficiency of overcoming disease, while experience of being joyful lead to healthy baby growth and stronger immune system. Epigenetic changes thus also lead to helpful outcomes, e.g., can increase the immunity of an individual. The primary epigenetic changes are DNA methylation, chromatin modification and acetylation which plays an important role in learning and memory formation. Interaction of an individual with diverse environmental experiences can influence the modulation of brain cells. Different interactions usually alter the DNA methylation status in brain cells which alters memory formation in the brain (27). DNA methylation in neurons may vary according to the type and duration of interaction, thus causes diversity and variability in memory formation (27, 28). Certain brain related problems are associated with dysfunctional memory that may partly be due to altered epigenetic status in several genes causing their altered expression. The interesting part about epigenetic alterations is that they are uncertain and coincide with uncertainties in quantum mechanics. Thus, it is rightly said that epigenetics is the biology’s quantum mechanics by Jorgensen (29). The outcome of environmental interaction in brain could be circuit specific. Thus, finding a specific circuit, whose interaction with particular environmental stimulus brings up modulation in behaviour is a difficult task. This seems to be the tedious job and challenging task due to limited knowledge about brain. Learning how the brain perceives information from environment, the outcome of which causes massive changes in behaviour advance us towards better understanding of brain functions. In an organ like brain where there are billions of cells interconnected with trillions of connections, attempting to target any specific brain circuit among billions seems improbable. Thus, it is assumed that quantum mechanics can help to understand the target specificity better than Newtonian mechanics as it usually rely on holistic approach rather than reductional.

## Understanding in light of Quantum Mechanics

The onset of psychiatric disorders is largely associated with individual experiences and heterogeneity. Diverse kinds of individual interactions with environment lead to the modulation of brain cells giving rise to epigenetic alteration. Thus brain disorders could be highly personalised with wide differences in their developmental pathways. With this contention, it can be said that development of brain disease seems following the equations of mathematics and calculus. The pattern in which a given problem may develop by more than one way and can also be solved by multiple ways. For example, in a scale of 0–10, if any marked disorder is represented by 10, several ways can be deduced to reach 10 like 0+1+2+3+4=10, 0+3+2+5=10 or 0+4+6=10. Such phenomenon when associated with quantum probabilities turns even more complex. In quantum mechanics, different particles have probability of being present at one place or a single particle may be present at more than one place at any given time. Thus, it can be assumed that in case of paranormal phenomenon, subatomic structures in brain and the associated consciousness could be involved in activating multiple awareness’s at once. Such awareness in certain cases might turn into delusions as seen in several psychiatric diseases like bipolar depression and schizophrenia. This seems quite similar to symptoms of paranormal phenomenon associated with several psychiatric disorders. Further, the limitations with current drugs include their inability to target multiple pathways which are involved in the development of symptoms. We do not understand yet, how and why there is a huge diversity in gene and environment interaction even between same phenotypes. Monozygotic twins or individuals sharing same environment and later develop variable epigenetic alterations are important examples. Epigenetic alterations are usually the chemical modifications causing changes in methylation which are often referred as methyl epimarks. These epimarks are developed due to interactions with the environment. The epigenetic modifications can be reversed by removing the epimarks or tags which causes behavioural changes (30, 31). As environmental experiences differ widely between individuals, a single risk factor could lead to different outcomes. This further complicates the effect of drugs on different individuals. It is assumed that such variations in living organisms including that of psychiatric disorders advances through mathematical equations. A question can be asked here, do current therapeutic drugs have potential to reverse such equations? If not then, in order to develop such drugs, we must first explore such equations by which abnormal behaviour is assumed to develop. This is exemplified here for example, if a teacher asks a group of students to solve certain type of calculus equation, the probable chances are each students may prefer his own way. Thus, in similar way, it is assumed that often in the development of any disease, there are more than one mechanism. Now the question is how could we learn and decode about the individual differences in the development of any disease. Currently, we have no answer to such questions due to our limited perspective on brain sciences. It is assumed that something specially personalised like consciousness must also be the part of this process. Due to reversible nature of epigenetics and the dynamic mechanisms involved in brain plasticity, there are wide variations in behaviour (23). A premier need is to understand the environment and its relation to epigenetic alteration. The environment and its associated risk factors have massive role in triggering the onset of disease. The important thing, however is, we must need to understand what environment is, and how it integrates into genome. Understanding the gene environment interaction has massive implications over how we proceed towards the treatment of diseases.

## Brain Versus Quantum Mechanics

In wide range of biological functions taking place in neurons and other eukaryotic cells, it is predicted that quantum effects are mediated by ubiquitous presence of microtubules and also have involvement in cognitive and regulatory functions (32). The coherence in photons can be due to the optical paths which act as waveguides of microtubules. Microtubules which are spatially distributed over hundreds of microns is said to have superposition of states which is strengthened in their interior due to the quantum coherence. Further computations and more specific biological functions have been associated with quantum coherence. Common bimolecular interactions like neurotransmitter receptor protein self assembly, enzyme substrate, antigen antibody reactions are facilitated by a superposition of spatially separated electron states (33). The ability of the human brain to produce neural oscillations in cooperative and coordinated manners by integrating several functional parts that provide basis for conscious activity is referred as metastability. Metastability of the brain has been proposed as one principle responsible for the brain and behavioural functions (34). The microtubules which are in superposition to other microtubules hundreds of microns away with different directions lead to quantum dynamics in microtubules over brain wide areas. Thus it was assumed earlier that unity of thought and consciousness arises due to the superposition over brain wide areas (32). The existence of consciousness can be viewed as the emergent property of physical systems (12). In brain, the cytoskeletal microtubules within neurons were proposed as possible sites for quantum effects (12). Linoleic acid is an important fatty acid concentrated in brain and is responsible for several psychopathological conditions due to its altered levels. It is said that this fatty acid and neural cell membrane acts as “key that open to the quantum behaviour” through the dynamics of cytoskeleton (35).

The fundamental aspect of quantum theory is wave function duality of atoms and their components. The important character exhibited by the subatomic particles is based on quantum character. Subatomic particles behave as wave of possibilities, however when observed by a conscious observer, they undergo collapse in their wave function and appears as particle. Thus, state of the quantum particles can be altered upon observation by conscious observer. It has observed that mental processes and mindful thoughts when focussed upon can alter the outcome, thus seems to have some common basis with quantum mechanics. With such observations, the duality of the mind/brain and that of wave/particle were presented with broad similarities (36).

What causes the wave function collapse due to conscious mind and confers solidity is not known. The greatest surprise from quantum theory comes in the form of quantum coherence, also called as quantum entanglement. Quantum coherence is the basis of all living organisation and accounts for the key features of conscious experience (37). It was observed first by Schrodinger in 1935, that wave functions of two quantum systems upon interaction become phase entangled. Thus the collapse of the wave function of one quantum system leads to the instantaneous wave function collapse of the other system, irrespective of how much the two systems are far apart (38). Quantum theory implies the surprising property of quantum inseparability which states that objects which have been once interacted are in same sense still connected even after being apart. This inseparability of quantum systems has been proved several times in past (39, 40). Recently, a neural model has been proposed about how brain stores memory and processes quantum information. This model could be employed for glutamatergic neurotransmission based quantum processing that also includes specific biochemical components (41). In addition, several techniques like Transcranial Magnetic Stimulation (TMS) which is important therapeutics is effectively used against variety of pathological condition like depression, stroke, pain, migraine etc. The possible mechanisms by which TMS operate are its associated quantum effects, the magnetic spin effect, genetic magneto reception etc (42). Recently posner molecules Ca_9_(PO_4_)_6_ are been identified as the unique molecules serving as working quantum memory. These molecules are believed to protect the neural qubits over a long time. Further chemical binding and subsequent melting of a pair of posner molecules results in release of shower of calcium ions that triggers neurotransmitter release, thus helps to enhance the neuron firing (43).

Importantly nowadays the emphasis is given on using models for understanding quantum cognition and decision making. An alternative probabilistic frame work offered by the quantum theory for modelling decision making has been successfully used to address behaviour related paradoxes (44–47).

Recent research suggests that the DNA conformation can be modulated due to heart focussed intentions (48). It is said that connections exist between the DNA of our cells and higher dimensional structures which are maintained in the quantum vacuum and organised in holographic fashion. These connections are highly energetic leading to coupling of information via resonance mechanisms. Further, it is suggested that energetic connections exist between the quantum vacuum through its structures which are connected to the corresponding structures in physical planes and can be influenced by humans intentionally. Thus, intentions which play a role on our strong faith, prayers with positive effects, placebo effects-a well acknowledged but poorly understood phenomenon contributes towards the better health and healing from serious disease. The intentions and association between heart and brain has a significant role in maintaining the overall health of individual.

## Conclusion

It is clear that earlier understanding of biological processes, including brain and its functions is mostly based on Newtonian physics. Such understanding based on classical physics, escapes our rationale about wide range of living functions, more importantly the functions of brain. With the advent of quantum mechanics, it has been observed time and again that the functions of the brain and principles of quantum mechanics coincide to a greater extent. Further, as though epigenetic alterations like DNA methylation and chromatin modification has important role in learning and memory formation. These epigenetic changes also face uncertainty like that of quantum mechanics events. For example, which specific gene at which particular location by which environmental stimulus with how much interactive time undergoes change is unpredictable? Further, as psychiatric disorders are usually associated with the dysfunctions in cognition, unpredictable epigenetic changes also add to their complexity. Thus, as the brain undergoes some unnatural phenomenon like experiencing delusional and paranormal symptoms, they turn quite similar to what we comprehend as the fundamental nature of quantum subatomic particles. We just do not know much about how brain works, though in the last few decades we begin to understand that principles of several brain functions are sharing the parallel phenomenon with quantum mechanics. Thus, these similarities force us to think again, that understanding the neurobehavioural complexity propels us to bring neurobehaviour and quantum mechanics more close than ever before.

## References

[1] Agid Y, Buzsáki G, Diamond DM, Frackowiak R, Giedd J, Girault JA (2007). How can drug discovery for psychiatric disorders be improved?. Nature Reviews Drug Discovery.

[2] Merz KM (2014). Using quantum mechanical approaches to study biological systems. Acc Chem Res.

[3] Perlovsky LI (2016). Physics of the mind. Front Syst Neurosci.

[4] Schoeller F, Perlovsky L, Arseniev D (2018). Physics of mind: experimental confirmations of theoretical predictions. Physics of Life Reviews.

[5] Schwartz JM, Stapp HP, Beauregard M (2005). Quantum physics in neuroscience and psychology: a neurophysical model of mind–brain interaction. Phil Trans R Soc B.

[6] Wachter A (2010). Relativistic quantum mechanics.

[7] Capra F (1996). The web of life: a new scientific understanding of living systems.

[8] Burtt EA (2016). The metaphysical foundations of modern science.

[9] Baily C, Finkelstein ND (2010). Teaching and understanding of quantum interpretations in modern physics courses. Phys Rev ST Phys Educ Res.

[10] Cohen-Tannoudji C, Guéry-Odelin D (2011). Advances in atomic physics: an overview [Internet].

[11] Hameroff S, Penrose R (2014). Consciousness in the universe: a review of the ‘Orch OR’ theory. Phys Life Rev.

[12] Hameroff SR (1994). Quantum coherence in microtubules: a neural basis for emergent consciousness?. J Conscious Stud.

[13] Rounsaville BJ, Weissman MM, Kleber H, Wilber C (1982). Heterogeneity of psychiatric diagnosis in treated opiate addicts. Arch Gen Psychiatry.

[14] Tessier-Lavigne M, Goodman CS (1996). The molecular biology of axon guidance. Science.

[15] Rosenzweig MR, Bermúdez-Rattoni F (2007). Modification of brain circuits through experience. Neural plasticity and memory: from genes to brain imaging.

[16] Stahl SM (2012). Psychotherapy as an epigenetic ‘drug’: psychiatric therapeutics target symptoms linked to malfunctioning brain circuits with psychotherapy as well as with drugs. J Clin Pharm Ther.

[17] Wani AL, Ara A (2015). Gene environment meshing: a primordial stepping towards behavioural modulation. Postępy Psychiatrii i Neurologii.

[18] Cara E (2014). The most dangerous idea in mental health. [Internet].

[19] Kinderman P (2014). Why we need to abandon the disease model of mental health care. Scientific American [Internet].

[20] Hoehe MR, Church GM, Lehrach H, Kroslak T, Palczewski S, Nowick K (2014). Multiple haplotype-resolved genomes reveal population patterns of gene and protein diplotypes. Nat Commun.

[21] Wani AL (2015). Personalized medicine: a near future or yet miles to go?. Advances in Integrative Medicine.

[22] Pauli W (1955). The influence of archetypal ideas on the scientific theories of Kepler. The interpretation of nature and the psyche.

[23] Kolb B, Gibb R, Robinson TE (2003). Brain plasticity and behavior. Curr Dir Psychol Sci.

[24] Doidge N (2007). The brain that changes itself.

[25] Schwartz JM, Begley S (2002). The mind and the brain: neuroplasticity and the power of mental force.

[26] Pennisi E (2001). Behind the scenes of gene expression. Science.

[27] Day JJ, Sweatt JD (2010). DNA methylation and memory formation. Nat Neurosci.

[28] Wani AL (2017). Understanding adult neurogenesis beyond its role in learning and memory formation. Educación Médica.

[29] Jorgensen RA (2011). Epigenetics: biology’s quantum mechanics. Front Plant Sci.

[30] Gottesman II, Hanson DR (2005). Human development: biological and genetic processes. Annu Rev Psychol.

[31] Campbell IC, Mill J, Uher R, Schmidt U (2011). Eating disorders, gene-environment interactions and epigenetics. Neurosci Biobehav Rev.

[32] Jibu M, Hagan S, Hameroff SR, Pribram KH, Yasue K (1994). Quantum optical coherence in cytoskeletal microtubules: implications for brain function. BioSystems.

[33] Conrad M (1992). Quantum molecular computing: the self-assembly model. Int J Quantum Chem.

[34] Kelso JAS, Tognoli E, Perlovsky LI, Kozma R (2007). Toward a complementary neuroscience: metastable coordination dynamics of the brain. Neurodynamics of higher-level cognition and consciousness.

[35] Cocchi M, Minuto C, Tonello L, Gabrielli F, Bernroider G, Tuszynski JA (2017). Linoleic acid: is this the key that unlocks the quantum brain? Insights linking broken symmetries in molecular biology, mood disorders and personalistic emergentism. BMC Neuroscience.

[36] Pullman B, Pullman A (1963). Quantum biochemistry.

[37] Ho MW (1997). Quantum coherence and conscious experience. Kybernetes.

[38] Herbert N (1993). Elemental mind, human consciousness and the new Physics.

[39] Clauser JF, Horne AH, Shimony A, Holt RA (1969). Proposed experiment to test local hidden variable theories. Phys Rev Lett.

[40] Aspect A, Grangier P, Wheeler JA, Zurek WH (1986). Experiments on Einstein-Podolsky-Rosen-type correlations with pairs of visible photons. Quantum theory and measurement.

[41] Weingarten CP, Doraiswamy PM, Fisher MPA (2016). A new spin on neural processing: quantum cognition. Front Hum Neurosci.

[42] Chervyakov AV, Chernyavsky AY, Sinitsyn DO, Piradov MA (2015). Possible mechanisms underlying the therapeutic effects of transcranial magnetic stimulation. Front Hum Neurosci.

[43] Fisher MPA (2015). Quantum cognition: the possibility of processing with nuclear spins in the brain. Ann Phys.

[44] Yearsleya JM, Busemeyer JR (2016). Quantum cognition and decision theories: a tutorial. J Math Psychol.

[45] Busemeyer JR, Wang Z (2015). What is quantum cognition, and how is it applied to psychology?. Curr Dir Psychol Sci.

[46] Pothos EM, Busemeyer JR (2013). Can quantum probability provide a new direction for cognitive modeling?. Behav Brain Sci.

[47] Pothos EM, Busemeyer JR, Trueblood JS (2013). A quantum geometric model of similarity. Psychol Rev.

[48] Mccraty R, Atkinson A, Tomasino D (2003). Modulation of DNA conformation by heart-focused intention.

